# Genetic Variations and Diseases in UniProtKB/Swiss-Prot: The Ins and Outs of Expert Manual Curation

**DOI:** 10.1002/humu.22594

**Published:** 2014-06-24

**Authors:** Maria Livia Famiglietti, Anne Estreicher, Arnaud Gos, Jerven Bolleman, Sébastien Géhant, Lionel Breuza, Alan Bridge, Sylvain Poux, Nicole Redaschi, Lydie Bougueleret, Ioannis Xenarios

**Affiliations:** 1 Swiss-Prot Group, SIB Swiss Institute of Bioinformatics, Centre Medical UniversitaireGeneva, Switzerland; 2 Vital-IT Group, SIB Swiss Institute of Bioinformatics, Quartier Sorge, Bâtiment GénopodeLausanne, Switzerland; 3 Center for Integrative Genomics, Quartier Sorge, University of LausanneLausanne, Switzerland; 4 European Molecular Biology Laboratory, European Bioinformatics Institute (EMBL-EBI), Wellcome Trust Genome Campus, HinxtonCambridge, UK; 5 Protein Information Resource, Georgetown University Medical CenterWashington, DC; 6 Protein Information Resource, University of DelawareNewark, Delaware

**Keywords:** UniProtKB/Swiss-Prot, database, manual curation, genetic variants, disease, functional annotation, controlled vocabulary

## Abstract

During the last few years, next-generation sequencing (NGS) technologies have accelerated the detection of genetic variants resulting in the rapid discovery of new disease-associated genes. However, the wealth of variation data made available by NGS alone is not sufficient to understand the mechanisms underlying disease pathogenesis and manifestation. Multidisciplinary approaches combining sequence and clinical data with prior biological knowledge are needed to unravel the role of genetic variants in human health and disease. In this context, it is crucial that these data are linked, organized, and made readily available through reliable online resources. The Swiss-Prot section of the Universal Protein Knowledgebase (UniProtKB/Swiss-Prot) provides the scientific community with a collection of information on protein functions, interactions, biological pathways, as well as human genetic diseases and variants, all manually reviewed by experts. In this article, we present an overview of the information content of UniProtKB/Swiss-Prot to show how this knowledgebase can support researchers in the elucidation of the mechanisms leading from a molecular defect to a disease phenotype.

## Introduction

During the past decade, the widespread application of next-generation sequencing technologies [Shendure and Ji, 2008] to the study of human populations has accelerated the rate of identification of human genetic variants [1000 Genomes Project Consortium et al., 2012; Tennessen et al., 2012], although establishing causal relationships between variants and disease phenotypes remains a major challenge. Multidisciplinary approaches that combine patient genomic sequence and clinical data with prior biological knowledge [Davies et al., 2012] are one means to fully elucidate disease mechanisms and establish the contribution of individual genetic variations to the development and progression of human diseases [Barabási et al., 2011; Furlong, 2013]. Although disease mutations could in principle occur in any functional region of the genome, most recent studies have utilized exome sequencing technologies to identify those affecting protein-coding regions (see, for instance, the NHLBI exome sequencing project at http://evs.gs.washington.edu/EVS/). In this context, high-quality resources linking genetic and medical information to protein sequences and associated biological knowledge, such as the manually curated section of the Universal Protein Knowledgebase (UniProtKB/Swiss-Prot), may be extremely valuable. UniProtKB/Swiss-Prot provides the scientific community with a collection of functional information on proteins, with accurate, consistent, and rich annotations, all manually reviewed by experts. Each UniProtKB/Swiss-Prot entry contains not only manually annotated protein sequence(s) encoded by one gene, but also expert curated functional annotations, mostly gathered from the scientific literature.

A complete, expertly curated human proteome was first made publicly available in UniProtKB/Swiss-Prot in September 2008. By “complete”, we mean that at least one representative protein sequence has been reviewed and annotated by an expert curator for each protein-coding gene. This complete human proteome has since been continuously reviewed and updated at each release, and currently consists of approximately 20,300 entries, representing a total of some 40,000 sequences (including alternative splicing isoforms). The process of expert manual curation includes a thorough review of available information on sequence variants (mostly single amino acid polymorphisms (SAPs)), and associated genetic disease information, as well as the normal protein function. Close to 70,000 SAPs are currently reported in UniProtKB/Swiss-Prot, 35% of which are associated with one of over 4,000 described genetic diseases. UniProtKB/Swiss-Prot stores information on variants along with functional data, structural information, protein–protein interaction data, pathways, and phenotypic descriptions. This global view can help users infer or establish relationships between variants and disease phenotypes.

The aim of this article is to show users how to best exploit the knowledge on protein function and sequence variation provided by UniProtKB/Swiss-Prot for the generation of new hypotheses describing the mechanisms underlying genetic diseases.

## Sequences and Variations

Variant annotation in UniProtKB/Swiss-Prot is the result of critical reading of relevant articles combined with an in-depth sequence analysis. In order to provide a reliable set of protein variants, the first step is to provide a correct sequence that could serve as a reference for subsequent variant description. This process involves the creation of a “canonical” sequence, which is displayed by default in the entry. Our policy is to have the canonical sequence matching the translation of the reference genome. This sequence is often the longest available, since this allows the description of the largest amount of features. Any discrepancy between the canonical sequence and others reported either in the literature, or in submissions to the International Nucleotide Sequence Database Collaboration (INSDC, i.e. DDBJ, ENA, and GenBank), is thoroughly reported in “Sequence annotation (Features)” in the appropriate subsection. These differences may be biologically relevant, such as alternative promoter usage, alternative splicing, alternative translation initiation, genetic variants, RNA editing, or technical artifacts, such as sequencing errors. To discriminate between these alternatives, sequences are thoroughly studied; this involves the analysis of the gene structure, a comparison with orthologous sequences and available Expressed Sequence Tags (ESTs), as well as searches for published experimental data. Alternative splicing isoforms are reported in such a way that at least one isoform (canonical or alternative) described in the entry matches a consensus CDS (http://www.ncbi.nlm.nih.gov/CCDS/). Genetic variants stored in UniProtKB/Swiss-Prot correspond to missense changes and small insertion–deletions (indels) that have either been reported in peer-reviewed publications, or are supported by several cDNAs and submitted to dbSNP, a database that catalogs short variations in nucleotide sequences. Mutations that truncate or grossly alter the protein sequence (such as frameshifts and nonsense mutations) are not currently annotated, but will be annotated in the near future.

## The Process of Expert Variant Curation

**Figure 1:**
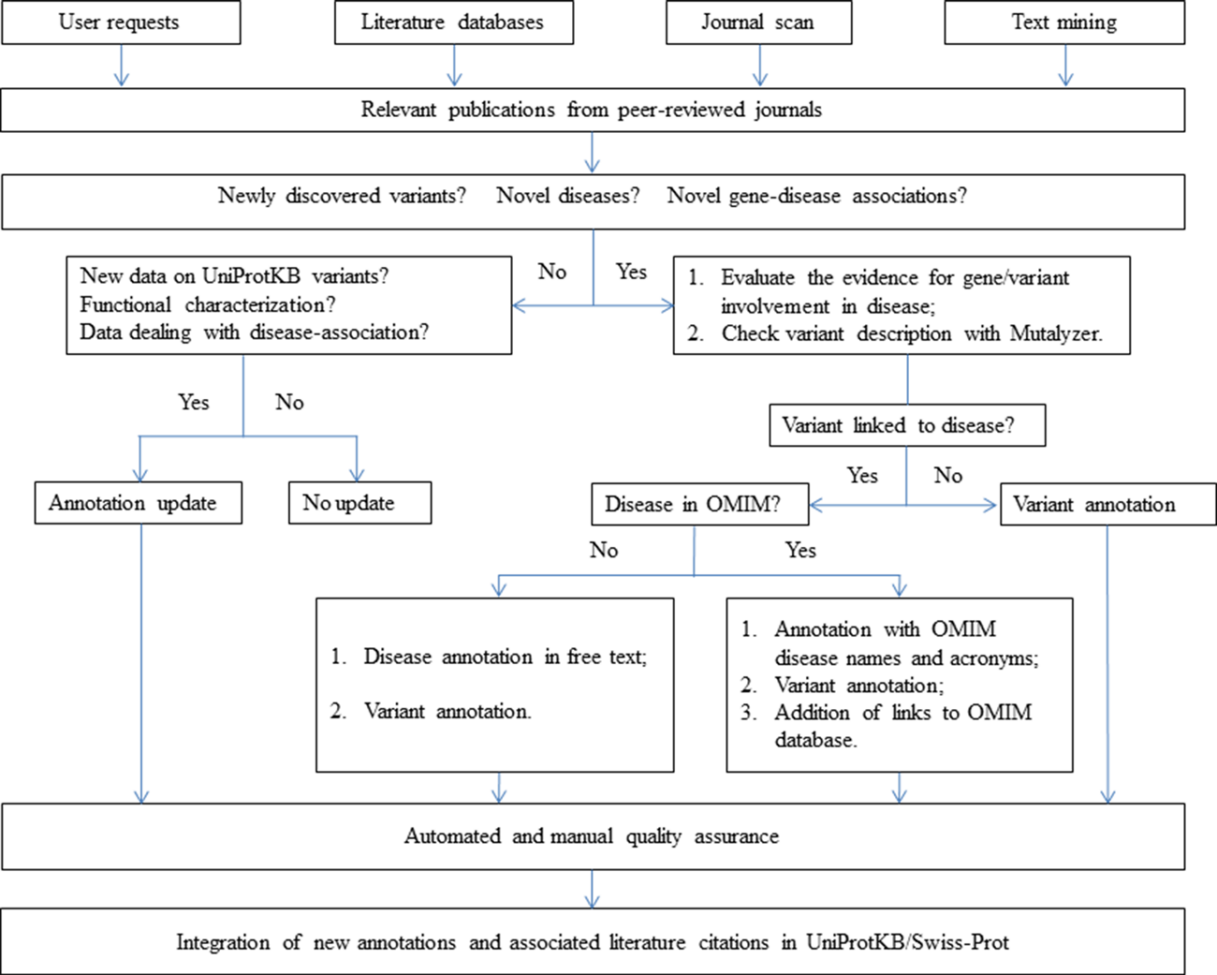
Flowchart of variant annotation in UniProtKB/Swiss-Prot. Variant annotation is based on published experimental data. The first step is to select a relevant article. This is achieved using several complementary approaches, including browsing specialized journals, alerts from literature databases and text mining. Users are also invited to take part in this process by contacting us to draw our attention on obsolete entries and/or to interesting publications. Articles linking protein information with medical disorders are critically reviewed by expert curators, and variant identification, disease description, and/or protein functional characterization are annotated based on supporting evidence. This annotation is submitted to various manual and automated checks before final integration into UniProtKB/Swiss-Prot. The disease nomenclature is based on OMIM, if available. If the disorder is not reported in OMIM, names and acronyms are created by the UniProtKB/Swiss-Prot staff on the basis of published reports.

**Figure 2:**
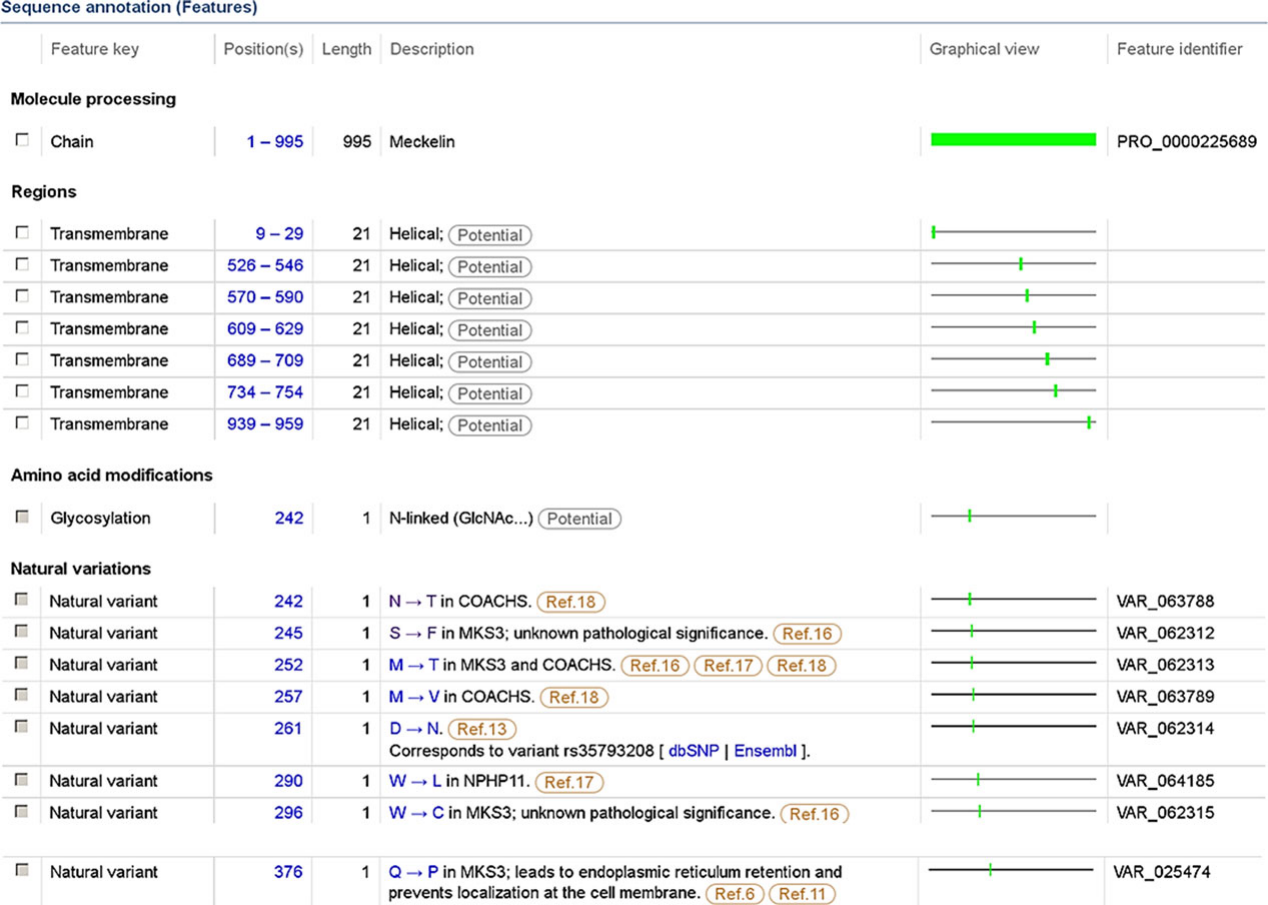
Excerpt from UniProtKB/Swiss-Prot entry Q5HYA8 representing human Meckelin (TMEM67). The “Sequence annotation (Features)” section describes the sequence and sequence variants at the single residue level. Note the presence of three types of variants: a neutral polymorphism at position 261, disease variants associated with ciliopathies MKS3, COACHS, and NPHP11, and VUS at positions 245 and 296. Note that disease-linked variant p.Asn242Thr affects a predicted N-glycosylation site (see subsection “Amino acid modifications”). Disease-linked variant p.Gln376Pro perturbs protein subcellular location.

Expert curation is a costly and time-consuming endeavor. At UniProtKB, we focus our curation efforts on gathering information from peer-reviewed literature that deals with disease-associated mutations with an impact on protein function. Neutral polymorphisms and variants of unknown pathological significance (VUS), i.e. whose association with disease or disease risk is unknown, can also be reported. The curation process (Fig.1) starts with the selection of relevant publications that describe the identification of new variant(s) and/or novel gene–disease associations, the functional characterization of new or existing variants, or that provide new information on protein function. A text-mining tool has been developed to improve this process [Veuthey et al., 2014, submitted]. Users can also contact us to draw our attention to particularly interesting publications that are missing in the knowledgebase. Once a publication has been selected for annotation, the whole text is critically read and pertinent information is reported in UniProtKB/Swiss-Prot as described elsewhere [Poux et al., 2014]. All genetic variants to be curated, be it neutral polymorphisms, disease-associated mutations, or unclassified variants, are controlled with the Mutation Analyzer (Mutalyzer) sequence variation nomenclature checker [Wildeman et al., 2008], using the reference sequence cited by the authors. If the variant is correctly designated and the variant description at nucleotide level is consistent with the proposed change at protein level, it is integrated into UniProtKB/Swiss-Prot in “Sequence annotation (Features)”, “Natural variant” (Fig.2). However, this position in UniProtKB/Swiss-Prot may not be identical to that published, since the position depends upon the reference sequence used. The canonical sequence that serves as reference may differ from the published reference sequence, for example, they can be different alternative splicing isoforms. There may be other reasons for discrepancies between published positions and UniProtKB/Swiss-Prot. Some classical numbering systems may be a legacy from the past. For instance, the numbering of collagen alpha-1(I) chain (COL1A1) (UniProtKB/Swiss-Prot accession P02452) variants traditionally starts at the beginning of the triple-helical region at position 179 (when position 1 corresponds to the initiator methionine), variants located in the N-terminus are simply ignored. In UniProtKB/Swiss-Prot, COL1A1 variants, as well as all other sequence features, are always numbered based on the canonical sequence, starting at the initiator methionine. Each annotated variant is given a unique and stable identifier (FTId) to allow the implementation of reciprocal links with variation records in other databases and also to facilitate citations in publications. If the variant is reported in dbSNP, a link is provided.

If a variant is associated with one or more diseases, the acronym of the disease(s) is indicated in the description field in “Sequence annotation (Features)” (Fig.2). Disease phenotypes are described in “General annotation (Comments)” section, “Involvement in disease” (see below). The descriptions are concise and cover only the main phenotypic features. They first indicate the name of the disease, followed by its acronym, and, if available, a link to the OMIM database (http://www.omim.org/). Users can follow this link to get additional information. After the disease description, a note, written in controlled vocabulary, indicates the relationship between the gene product and the disease, whether it be disease causing, disease modifying, or altering the susceptibility to disease.

## UniProtKB/Swiss-Prot Variant Pages

**Figure 3:**
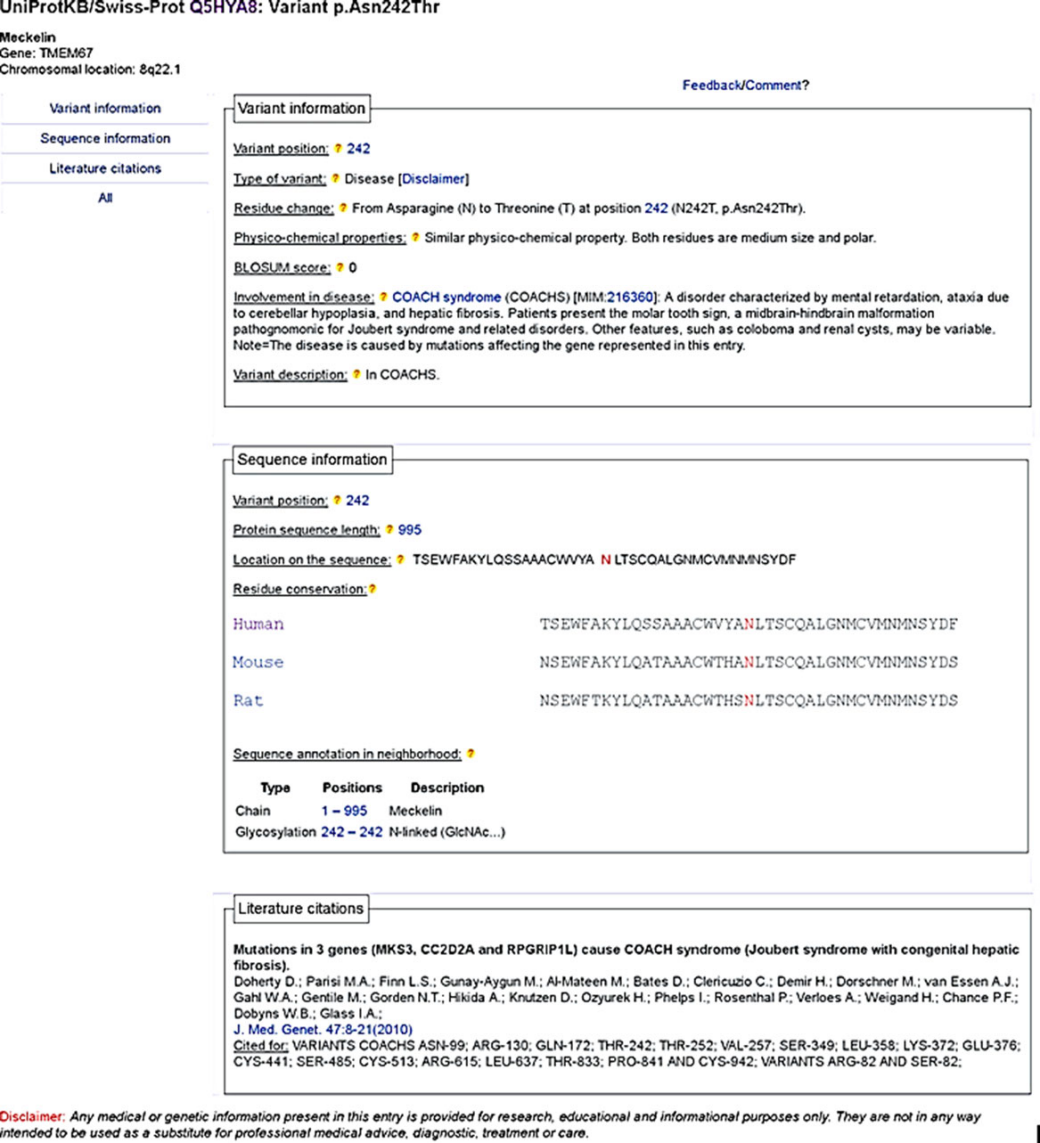
UniProtKB/Swiss-Prot page for human Meckelin (TMEM67) variant p.Asn242Thr.

The variant-specific annotations can be visualized in the UniProtKB/Swiss-Prot variant web pages [Yip et al., 2004]. These pages can be accessed directly from the description field in “Sequence annotation (Features)”. They contain the description of a defined variant, the physicochemical properties of the original residue and that of the missense, the BLOSUM score for the amino acid change, the conservation of the residue across various species, the regions, domains, or sites annotated in its vicinity (Fig.3). The potential association with a disease and the description of the phenotype are also reported, as well as the bibliographic reference(s) dealing with the variant.

## On the Importance of Linking Variation Data to Functional Characterization Data

In the last few years, it has become evident that genotype–phenotype relationships are not straightforward [Cooper et al., 2013]. As shown by recent studies [Das et al., 2014; Kenna et al., 2013; Piton et al., 2013], variants originally reported as disease-associated may need to be reclassified as benign polymorphisms, and vice versa, variants thought to be neutral polymorphisms may be reassessed as contributors to disease when more sequencing and genotyping data of both disease and apparently healthy populations become available. Variant classification is an error-prone exercise whose result depends on the amount and quality of available data. An efficient approach to the understanding of the pathological relevance of genetic variants should take into account experimental evidences of variant effect on protein properties, as a complement to statistical genetics considerations and computational methods predicting missense variants deleteriousness. Thanks to rich and accurate annotations, variant data in UniProtKB/Swiss-Prot are embedded in the biochemical context defining protein properties and activities. Whenever variant characterization data are available in the literature, it is reported in the description field in “Sequence annotation (Features)”, “Natural variant” (Fig.2), in free text form. Currently, about 6,200 variants in UniProtKB/Swiss-Prot are associated with some curated characterization data, and this number is continuously increasing. We are also working on improving the representation of this information by combining existing ontologies such as the Variation Ontology (VariO) (http://www.variationontology.org), which describes variants and their effects [Vihinen, 2014], and the Gene Ontology (GO), which describes the characteristics of normal proteins (and which is already used routinely in UniProtKB). A combination of terms from the two ontologies is used to specify the characteristic of a normal protein (GO) and the effect of a given variant on it (VariO).

To further facilitate data access and utilization, a new interface that provides powerful SPARQL queries on the Resource Description Framework (RDF) representation of curated variant data from UniProtKB/Swiss-Prot is being developed. This will allow users to perform complex queries to search for variants occurring at known functionally relevant sites (query examples are provided in the Supporting Information).

Because of their reliability, UniProtKB/Swiss-Prot annotations have also been used to develop prediction methods to estimate the pathogenicity of missense variants [Thusberg et al., 2011; Capriotti et al., 2013; Shihab et al., 2013; Yates and Sternberg, 2013].

## Involvement in Disease

A good example to highlight the richness of UniProtKB/Swiss-Prot annotations is provided by proteins associated with ciliopathies, a group of diseases caused by abnormal cilia formation or function [Yuan and Sun, 2013]. Ciliopathies cover a large spectrum of often overlapping phenotypes [Waters and Beales, 2011]. The clinical overlap between different ciliopathies and their variable penetrance and expressivity are due to multilocus allelism with clinical outcome depending on the global mutational load in genes involved in cilia biology [Beales et al., 2003; Khanna et al., 2009; Davis et al., 2011]. Successful identification of gene mutations involved in ciliopathies has shown the role of disease-causing and disease-modifying genes, as well as gene–gene interactions, emphasizing the complexity of biological and pathological events.

**Figure 4:**
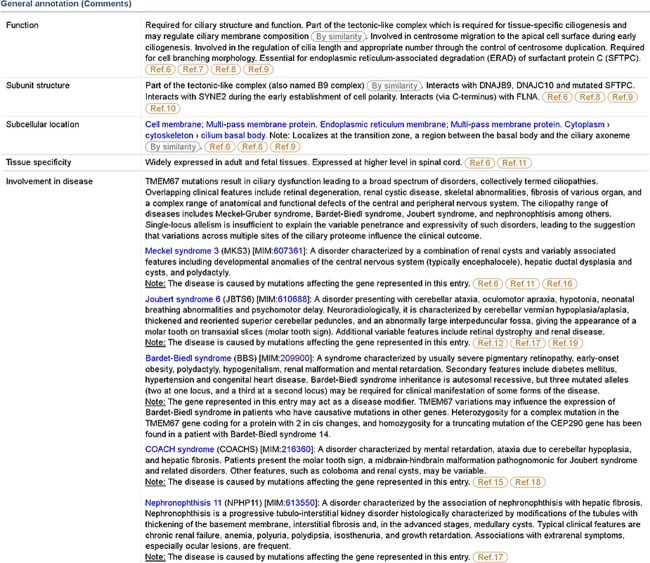
Excerpt from the “General annotation (Comments)” section in Q5HYA8, containing functional annotations based on publications. TMEM67 mutations are involved in several ciliopathies, including Meckel syndrome 3 (MKS3), Joubert syndrome 6 (JBTS6), Bardet–Biedl syndrome (BBS), COACH syndrome (COACHS), and nephronophthisis 11 (NPHP11). The precise type of association with the disease, i.e. confirmed or probable pathological role, susceptibility to disease or disease modification, is indicated in the “Note” using a controlled vocabulary.

For example, mutations in the TMEM67 gene (UniProtKB/Swiss-Prot accession Q5HYA8) have been shown to cause Meckel syndrome 3, Joubert syndrome 6, COACH syndrome, and nephronophthisis 11. This information is reported in the “Note” of the “Involvement in disease” subsection using the standardized expression “The disease is caused by mutations affecting the gene represented in this entry”. However, TMEM67 has been also associated with Bardet–Biedl syndrome, where it probably acts as a modifier. This information is annotated in the “Note” as “The gene represented in this entry may act as a disease modifier” (Fig.4). The note may also contain additional interesting information written in free text, such as explanations of the pathological mechanism at molecular level. For example, TRIM32 (UniProtKB/Swiss-Prot accession Q13049) has been shown to cause Bardet–Biedl syndrome 11 (BBS11). The role of TRIM32 in BBS11 pathogenesis is not obvious, since it is not directly involved in cilia function, assembly, or maintenance. It is an E3 ubiquitin ligase that may act by degrading BBS2, a component of a large complex involved in ciliary membrane biogenesis [Zhang et al., 2012]. Although it is a mere hypothesis for the time being, it gives a hint about the molecular mechanism that may underlie BBS11 pathogenesis. As such, it is reported in the second part of the note.

## Disease Nomenclature

When a disease is reported in the OMIM database, the OMIM disease name and acronym are imported into the UniProtKB documentation and used for annotation. This ensures consistency between both databases. This nomenclature may sometimes differ from that found in the medical literature. For example, the ciliopathy currently named “Joubert syndrome” in OMIM has also been called “cerebello-oculo-renal syndrome”, “cerebellooculorenal syndrome”, “cerebelloparenchymal disorder”, or “Joubert–Boltshauser syndrome” in various publications. Although in UniProtKB/Swiss-Prot entries, we only indicate the current name “Joubert syndrome” as proposed by the OMIM database, we provide an exhaustive list of disease synonyms in the humdisease.txt file (see below). Diseases that are not reported in OMIM are named by the UniProt curator staff on the basis of published reports.

## Retrieving Disease-Associated Proteins from UniProtKB/Swiss-Prot

A convenient way to retrieve proteins of interest from UniProtKB is to use appropriate keywords. In UniProtKB/Swiss-Prot, most keywords are attributed in the course of manual annotation procedure. They provide a summary of the entry content and can be used to generate indexes of protein entries based on functional, structural, or other categories such as “Cellular component,” “Coding sequence diversity,” and “Disease.” The category “Disease” contains over 150 keywords that are assigned to proteins involved in a disease. In general, UniProtKB/Swiss-Prot curators create new medical keywords when at least two proteins are involved in a specific disorder. Proteins that have been shown in the medical research literature to be associated with diseases characterized by either abnormal formation or function of cilia can be retrieved using the keyword “Ciliopathy”. The term “Ciliopathy” covers a wide variety of syndromes that can be associated with more than one defective protein. To allow targeted searches, more specific keywords have been created for defined types of ciliopathies, such as “Bardet–Biedl syndrome”, “Joubert syndrome”, and “Meckel syndrome”. Some keywords are associated with sequence features. For instance, proteins for which at least one disease-associated variant is described in “Sequence annotation (Features)”, “Natural variant”, can be retrieved using the keyword “Disease mutation”. The annotation of neutral variants or VUS into “Sequence annotation (Features)”, “Natural variant”, drives the addition of the keyword “Polymorphism”. UniProtKB keywords are stored in a controlled vocabulary list, available online in the UniProtKB documentation (keywlist.txt file).

## On the Importance of Linking Disease Descriptions to Protein Functional Annotations

Most genes identified so far as ciliopathy-causing factors encode proteins that are themselves components of the cilia or that are involved in cilium biogenesis, maintenance, and function. Information on the physiological protein function, its subcellular location, tissue expression, interaction with other proteins, enzymatic activity, etc., are stored in “General annotation (Comments)” (Fig.4). These annotations are classified in specialized subsections, some written in free text (“Function”, “Tissue specificity”, etc.) and some in controlled vocabulary (“Subcellular location”, “Catalytic activity”, etc.). Most controlled vocabulary subsections can contain a free text “Note =” that allows to qualify the annotation and provides additional information.

High-quality, detailed, and structured annotations may contribute to the discovery of gene–disease associations. For example, when a ciliopathy is mapped to a new locus and a list of genes at this locus is established, a quick glance at UniProtKB/Swiss-Prot may help to identify which ones are involved in ciliary biology, hence should be considered in priority as candidates for further analysis. This information can be easily found in “General annotation (Comments)”, “Function”, or searching with the keywords “Cilium”, for a protein found in, or associated with a cilium, or “Cilium biogenesis/degradation”, for proteins involved in the formation, organization, maintenance, and degradation of the cilium. Additionally, searches can be performed with GO terms related to cilia biology. Currently, in UniProtKB/Swiss-Prot, ∼150 proteins have been shown to be associated with cilium, either topologically or functionally, but have not been associated with any type of ciliopathy. These proteins could be considered for analysis in patients negative for mutations in any known ciliopathy genes. Alternatively, UniProtKB/Swiss-Prot could be convenient source for the identification of interaction partners, or as a central hub to access other specialized resources, such as IntAct (http://www.ebi.ac.uk/intact/) or STRING (http://string-db.org/).

## UniProtKB Genetic Disease and Variant-Dedicated Documents

Data on genetic diseases and variants are summarized in and distributed through the documents given below.

### Humdisease.txt

The controlled vocabulary used in UniProtKB/Swiss-Prot for the description of diseases is available in the file humdisease.txt in our documentation. This file deals only with diseases reported in the OMIM database. Diseases reported in UniProtKB/Swiss-Prot that do not have a counterpart in OMIM are written in free text, therefore are not represented in this file. The humdisease.txt file lists, for each disease, its name, acronym, synonyms, phenotypic description, and MeSH terms, and provides cross-references to the OMIM database. Disease names and acronyms are assigned according to the OMIM nomenclature, while synonym come from literature reports. Disease names and acronyms are used in “General annotation (Comments)”, “Involvement in disease”. Acronyms are used in “Sequence annotation (Features)”, “Natural variant”.

### Humsavar.txt

In release 2014_03 of March 2014, the total number of SAPs annotated in UniProtKB/Swiss-Prot was 68,908 (up-to-date statistics can be found on UniProtKB website). All are listed in the file humsavar.txt. This document also indicates if a SAP has been reported to be disease-associated (24,439 variants), a neutral polymorphism (37,904), or a VUS (6,565). The humsavar.txt file has been used as a reliable training set for the development of computational tools predicting the damaging effects of missense mutations [Care et al., 2007; Adzhubei et al., 2010].

### Homo_sapiens_variation.txt.gz

Variant annotation in UniProtKB/Swiss-Prot is focused on variations described in the literature. While high-quality expert curation is essential to support research, it is a slow process that cannot cope with the flood of data produced at increasing speed by new sequencing technologies. As a result, many variation data are missing from UniProtKB/Swiss-Prot. In order to provide users with a complete set of human variations, we have released a new extension to the humsavar.txt variant catalogue, the homo_sapiens_variation.txt.gz. This new file contains variants not annotated in UniProtKB/Swiss-Prot entries. It supplements the set of manually curated human variants in humsavar.txt with a catalogue of novel SAPs from the 1,000 Genomes Project. These variants have been automatically mapped to UniProtKB sequences, including isoform sequences, through Ensembl. In addition to defining the position and the amino acid change due to each variant, the new file maps each affected UniProtKB record to the corresponding Ensembl gene, transcript and protein identifiers, and provides the chromosomal location with allele change. Where possible, a cross-reference to OMIM is provided. This file, along with the humsavar.txt file, can be found in the new dedicated “variants” directory on the UniProt FTP site.

## Links to UniProt Website and Documents

http://www.uniprot.org/
http://www.uniprot.org/docs/keywlist
http://www.uniprot.org/docs/dbxref
http://www.uniprot.org/docs/humdisease
http://www.uniprot.org/docs/humsavar
ftp://ftp.uniprot.org/pub/databases/uniprot/current_release/knowledgebase/variants/

## UniProtKB/Swiss-Prot Data Integration with Other Resources

UniProtKB/Swiss-Prot has always striven to provide a high level of integration with other databases. The 129 databases that are currently cross-referenced in UniProtKB include nucleotide sequence archives (ENA, DDBJ, and GenBank), as well as databases dedicated to three-dimensional structures (experimental or predicted), interactions, and pathways. The “Cross-references” section also gives access to medically relevant databases, including DrugBank (http://www.drugbank.ca/), a resource that combines detailed drug data with comprehensive drug target, PharmGKB (https://www.pharmgkb.org/), a resource dealing with the impact of genetic variation on drug response, and Orphanet (http://www.orpha.net/), a free-access portal for information on rare diseases and orphan drugs. The latter database provides detailed clinical disease descriptions and terminologies. The full list of cross-references available from the “Cross-references” section and, in rare cases, “Sequence annotation (Features)” can be found in the dbxref.txt file.

A dedicated section called “Web resources” has been created to store links to locus-specific databases that, dealing with a single gene/disease, aim to record all gene variants identified worldwide. From this section, users can access information on variants not annotated in UniProtKB/Swiss-Prot.

## Conclusions

UniProtKB/Swiss-Prot is a freely accessible resource that offers concise and reliable information on proteins. This includes information on genetic, mostly disease-linked, variants at the protein level and a short description of the associated disease. The variant information is displayed in the general context of the whole sequence, side by side with the annotation of domains, post-translational modifications, and secondary structures. The phenotype resulting from protein alteration is described along with the physiological protein function, its subcellular location, tissue expression, etc. The integration of both physiological and pathological data may allow formulating new hypotheses on the molecular processes that lead to disease status.

In order to improve the clarity of disease information and to facilitate its retrieval from UniProtKB, the format of the subsection “Involvement in disease” is highly structured and written using standard phrases and controlled vocabulary. As clinical classifications and terminologies do not generally focus on genetic disease nomenclature, the disease naming system in UniProtKB follows the nomenclature provided by OMIM, a highly comprehensive knowledgebase of genetic syndromes. OMIM disease names are also used in the Human Phenotype Ontology (HPO) [Köhler et al., 2014], a resource that provides a controlled and structured vocabulary of phenotypic abnormalities encountered in human disorders. The consistency in disease naming in both UniProtKB/Swiss-Prot and HPO allows the mapping of UniProtKB sequences to HPO phenotypic terms.

The disease description found in “Involvement in disease” is a very concise summary from published observations. A detailed phenotypic description would be beyond the scope of a general interest protein knowledgebase, such as UniProtKB. Users interested by this kind of information should refer either to bibliographic citations stored in the entries or to cross-references to specialized databases. Among them is the Orphanet database, a portal for information on rare diseases. Rare disorders actually represent the majority of the diseases annotated in UniProtKB/Swiss-Prot. Orphanet maps these disorders to the International Classification of Diseases (ICD-10), SNOMED CT, MeSH, MedDRA, and UMLS [Rath et al., 2012]. In this way, cross-references to Orphanet link UniProtKB protein sequences to clinical terminologies.

UniProtKB/Swiss-Prot annotation is limited to disease-associated and/or experimentally characterized variants, mostly missense changes. The rationale is that UniProtKB is widely used in proteomics experiments as a primary database for protein identification. Many search engines used for mass spectrometry peptide identification take into account UniProtKB protein sequences, as well as all sequence variations annotated in the “Sequence annotation (Features)” section, including genetic variants. As missense variants represent a frequent type of variation, they have been the target of high-priority annotation. In this context, complex variants, such as those producing a premature stop codon, either directly or by altering splicing, are not relevant. However, this type of complex variants can be of crucial importance for geneticists that is why we are setting up a new format that will allow their representation in the near future.

In order to be useful to the scientific and medical community, the UniProtKB/Swiss-Prot SAP collection should be highly integrated with other resources. To be unambiguously identified, genetic variants are defined at the genomic DNA level. UniProtKB/Swiss-Prot is a protein knowledgebase and cataloguing variants at the DNA level is out of its scope. In order to bridge the gap, we have started to map UniProtKB/Swiss-Prot SAPs to RefSeq or Locus Reference Genomic (LRG) sequences [Dalgleish et al., 2010; Macarthur et al., 2014], and to submit them to central resources, such as dbSNP and Ensembl. A different approach has been taken for the integration of UniProtKB/Swiss-Prot variants into the University of California Santa Cruz (UCSC) genome browser. Here, UniProtKB/Swiss-Prot sequences were first aligned to RefSeq sequences and then UniProtKB/Swiss-Prot variants were lifted to genome positions with the pslMap program. This demonstrates that the integration of UniProtKB/Swiss-Prot data collection to genome browsers is not only possible, but is already achieved and made publicly available at the UCSC genome browser.

Expert curation is a time-consuming and expensive process, but it produces most reliable datasets. Improvement of dataset availability is a major objective in UniProtKB/Swiss-Prot variant annotation. This should be achieved by increasing integration with other resources and by facilitating parsing through the introduction of controlled vocabularies as much as possible.
