# Supplementary material for: Genetic Variations and Diseases in UniProtKB/Swiss-Prot: The Ins and Outs of Expert Manual Curation
Source: Hum Mutat. 2014 Jun 24;35(8):927–35. doi: 10.1002/humu.22594 (PMC4107114; doi:10.1002/humu.22594)
Supplement: Supplementary file 1 [file humu0035-0927-SD1.pdf]

Supporting Information: Query Examples for Human Diseases

Table of Contents

- Querying with SPARQL 1.1
- Number of annotations that overlap with a disease variant
- Number of annotation types that overlap with a disease variant
- Active site annotations that overlap with a disease variant
- Disulfide bond annotations that overlap with a disease variant

Querying with SPARQL 1.1

The main UniProt website http://www.uniprot.org is document centric to serve users who search for entries of their interest to browse them. It has limited support for analytical queries. To fill this gap, the UniProt consortium provides http://beta.sparql.uniprot.org for answering analytical queries and gather statistics on the UniProt data using SPARQL 1.1, a standardized query language that is resource centric.

All queries below where run against UniProtKB release 2014_01.

Number of annotations that overlap with a disease variant

```
PREFIX uniprotkb:<http://purl.uniprot.org/uniprot/>
PREFIX taxon:<http://purl.uniprot.org/taxonomy/>
PREFIX faldo:<http://biohackathon.org/resource/faldo#>
PREFIX up:<http://purl.uniprot.org/core/>
PREFIX rdf:<http://www.w3.org/1999/02/22-rdf-syntax-ns#>
PREFIX rdfs:<http://www.w3.org/2000/01/rdf-schema#>
PREFIX owl:<http://www.w3.org/2002/07/owl#>
SELECT (COUNT(?protein) as ?protCount)
WHERE
{
 ?protein a up:Protein ;
        up:organism taxon:9606 ;
        up:annotation ?variantAnnot , ?overlapAnnot , ?diseaseAnnot .
 ?variantAnnot a up:Natural_Variant_Annotation ;
            rdfs:comment ?variantComment ;
            up:range ?variantRange .
 ?variantRange faldo:begin/faldo:position ?variantBegin ;
            faldo:end/faldo:position ?variantEnd .
 ?diseaseAnnot a up:Disease_Annotation ;
            up:disease ?disease .
 ?disease up:mnemonic ?diseaseMnemonic .
 ?overlapAnnot a ?annotType ;
            up:range ?annotRange .
 ?annotRange faldo:begin/faldo:position ?annotBegin .
```

```
 OPTIONAL
 {
   ?annotRange faldo:end/faldo:position ?annotEnd .
 }
 FILTER(contains(?variantComment, ?diseaseMnemonic))
 FILTER((?annotBegin >= ?variantBegin && ?annotBegin <= ?variantEnd) || (?annotEnd >=
?variantBegin && ?annotEnd <= ?variantEnd))
 FILTER(!sameTerm(?annotType, up:Natural_Variant_Annotation))
}
```

There are 2722 annotations overlapping a disease related variant.

Number of annotation types that overlap with a disease variant

```
PREFIX uniprotkb:<http://purl.uniprot.org/uniprot/>
PREFIX taxon:<http://purl.uniprot.org/taxonomy/>
PREFIX faldo:<http://biohackathon.org/resource/faldo#>
PREFIX up:<http://purl.uniprot.org/core/>
PREFIX rdf:<http://www.w3.org/1999/02/22-rdf-syntax-ns#>
PREFIX rdfs:<http://www.w3.org/2000/01/rdf-schema#>
PREFIX owl:<http://www.w3.org/2002/07/owl#>
SELECT (COUNT(?protein) as ?protCount) ?annotTypeString
WHERE
{
 ?protein a up:Protein ;
          up:organism taxon:9606 ;
          up:annotation ?variantAnnot , ?overlapAnnot , ?diseaseAnnot .
 ?variantAnnot a up:Natural_Variant_Annotation ;
              rdfs:comment ?variantComment ;
              up:range ?variantRange .
 ?variantRange faldo:begin/faldo:position ?variantBegin ;
              faldo:end/faldo:position ?variantEnd .
 ?diseaseAnnot a up:Disease_Annotation ;
              up:disease ?disease .
 ?disease up:mnemonic ?diseaseMnemonic .
 ?overlapAnnot a ?annotType ;
              up:range ?annotRange .
 ?annotRange faldo:begin/faldo:position ?annotBegin .
 OPTIONAL
 {
   ?annotRange faldo:end/faldo:position ?annotEnd .
 }
 FILTER(contains(?variantComment, ?diseaseMnemonic))
 FILTER((?annotBegin >= ?variantBegin && ?annotBegin <= ?variantEnd) || (?annotEnd >=
?variantBegin && ?annotEnd <= ?variantEnd))
 FILTER(!sameTerm(?annotType, up:Natural_Variant_Annotation))
```

```
 BIND(substr(str(?annotType), 30) as ?annotTypeString)
}
GROUP BY ?annotTypeString ORDER BY ?annotTypeString
```

**Number of annotations**	**Type of annotation**
44	Active Site
70	Alternative Sequence
411	Beta Strand
122	Binding Site
4	Calcium Binding
23	Chain
10	Compositional Bias
3	Cross-link
643	Disulfide Bond
55	Domain Extent
4	Frameshift
52	Glycosylation
283	Helix
3	Intramembrane
3	Lipidation
7	Mass Spectrometry
126	Metal Binding
111	Modified Residue
18	Motif
266	Mutagenesis
28	NP Binding
1	Non-standard Residue
3	Nucleotide Binding
1	Peptide
3	Propeptide
57	Region
14	Repeat
85	Sequence Conflict
39	Site
82	Topological Domain
69	Transmembrane
77	Turn
5	Zinc Finger

Active site annotations that overlap with a disease variant

```
PREFIX uniprotkb:<http://purl.uniprot.org/uniprot/>
PREFIX taxon:<http://purl.uniprot.org/taxonomy/>
PREFIX faldo:<http://biohackathon.org/resource/faldo#>
PREFIX up:<http://purl.uniprot.org/core/>
PREFIX rdf:<http://www.w3.org/1999/02/22-rdf-syntax-ns#>
PREFIX rdfs:<http://www.w3.org/2000/01/rdf-schema#>
PREFIX owl:<http://www.w3.org/2002/07/owl#>
SELECT ?protein ?diseaseMnemonic ?annotBegin ?annotComment
WHERE
{
 ?protein a up:Protein ;
        up:organism taxon:9606 ;
        up:annotation ?variantAnnot , ?overlapAnnot , ?diseaseAnnot .
 ?variantAnnot a up:Natural_Variant_Annotation ;
          rdfs:comment ?variantComment ;
          up:range ?variantRange .
 ?variantRange faldo:begin/faldo:position ?variantBegin ;
          faldo:end/faldo:position ?variantEnd .
 ?diseaseAnnot a up:Disease_Annotation ;
          up:disease ?disease .
 ?disease up:mnemonic ?diseaseMnemonic .
 ?overlapAnnot a up:Active_Site_Annotation ;
          up:range ?annotRange .
 ?annotRange faldo:begin/faldo:position ?annotBegin ;
         faldo:end/faldo:position ?annotEnd .
 OPTIONAL { ?overlapAnnot rdfs:comment ?annotComment .}
 FILTER(contains(?variantComment, ?diseaseMnemonic))
 FILTER(?annotBegin >= ?variantBegin && ?annotBegin <= ?variantEnd)
}
```

Protein	Disease mnemonic	Active site position	Active site comment
P06280	FD	170	Nucleophile
P06280	FD	231	Proton donor
P03950	ALS9	138	Proton donor
O43520	BRIC1	454	4-aspartylphosphate intermediate
Q04656	MNKD	1044	4-aspartylphosphate intermediate
Q06187	XLA	521	Proton acceptor
Q06187	XLA	521	Proton acceptor
Q06187	XLA	521	Proton acceptor
O14773	CLN2	475	Charge relay system
P20807	LGMD2A	334	
P53634	PLS	405	

Protein	Disease mnemonic	Active site position	Active site comment
P53634	PLS	405	
P06276	BChE deficiency	226	Acyl-ester intermediate
P09417	HPABH4C	150	Proton acceptor
P00742	FA10D	322	Charge relay system
P03951	FA11D	575	Charge relay system
P00748	FA12D	461	Charge relay system
P08709	FA7D	302	Charge relay system
P08709	FA7D	302	Charge relay system
P08709	FA7D	404	Charge relay system
P00740	HEMB	411	Charge relay system
P00740	HEMB	411	Charge relay system
P35575	GSD1A	119	Proton donor
P06744	HA-GPID	389	
Q92947	GA1	414	Proton acceptor
P49773	NMAN	112	Tele-AMP-histidine intermediate
P35475	MPS1H	182	Proton donor
P04180	LCATD	205	Charge relay system
P06858	LPL deficiency	183	Charge relay system
P06858	LPL deficiency	183	Charge relay system
P06858	LPL deficiency	183	Charge relay system
P10253	GSD2	521	
P10253	GSD2	521	
P08253	MONA	404	
P34949	CDG1B	295	
Q99519	SIALIDOSIS	370	Nucleophile
P00480	OTCD	303	
P00480	OTCD	303	
P35913	RP40	557	Proton donor
Q9H237	FODH	341	
P60484	CWS1	124	Phosphocysteine intermediate
P60484	CWS1	124	Phosphocysteine intermediate
P00797	RTD	104	
Q9NUW8	SCAN1	493	Proton donor

Disulfide bond annotations that overlap with a disease variant

```
PREFIX uniprotkb:<http://purl.uniprot.org/uniprot/>
PREFIX taxon:<http://purl.uniprot.org/taxonomy/>
PREFIX faldo:<http://biohackathon.org/resource/faldo#>
PREFIX up:<http://purl.uniprot.org/core/>
PREFIX rdf:<http://www.w3.org/1999/02/22-rdf-syntax-ns#>
PREFIX rdfs:<http://www.w3.org/2000/01/rdf-schema#>
PREFIX owl:<http://www.w3.org/2002/07/owl#>
SELECT ?protein ?diseaseMnemonic ?annotBegin ?variantSubstitution
WHERE
{
 ?protein a up:Protein ;
        up:organism taxon:9606 ;
        up:annotation ?variantAnnot , ?overlapAnnot , ?diseaseAnnot .
 ?variantAnnot a up:Natural_Variant_Annotation ;
           rdfs:comment ?variantComment ;
           up:range ?variantRange ;
           up:substitution ?variantSubstitution .
 ?variantRange faldo:begin/faldo:position ?variantBegin ;
           faldo:end/faldo:position ?variantEnd .
 ?diseaseAnnot a up:Disease_Annotation ;
           up:disease ?disease .
 ?disease up:mnemonic ?diseaseMnemonic .
 ?overlapAnnot a up:Disulfide_Bond_Annotation ;
           up:range ?annotRange .
 OPTIONAL { ?overlapAnnot rdfs:comment ?annotComment .}
 ?annotRange faldo:begin/faldo:position ?annotBegin ;
          faldo:end/faldo:position ?annotEnd .
 FILTER(!(bound(?annotComment) && sameTerm(?annotBegin, ?annotEnd)))  #this removes
the interchain disulfidebonds
 FILTER(contains(?variantComment, ?diseaseMnemonic))
 FILTER(?annotBegin >= ?variantBegin && ?annotBegin <= ?variantEnd)
}
```

**Protein**	**Disease mnemonic**	**Disulfide bond position**	**Variant substitution**
P43026	AMDG	400	Y
P78504	ALGS1	187	S
P78504	ALGS1	187	Y
P78504	ALGS1	220	F
P78504	ALGS1	229	G
P78504	ALGS1	229	Y
P78504	ALGS1	271	R
P78504	ALGS1	284	F

**Protein**	**Disease mnemonic**	**Disulfide bond position**	**Variant substitution**
P78504	ALGS1	438	F
P78504	ALGS1	693	Y
P78504	ALGS1	714	Y
P78504	ALGS1	731	S
P78504	ALGS1	740	R
P78504	ALGS1	753	R
Q04721	ALGS2	444	Y
P00441	ALS1	147	R
P03950	ALS9	63	W
P01008	AT3D	127	R
P01008	AT3D	160	Y
P01008	AT3D	462	F
P21802	ABS2	342	R
P21802	ABS2	342	S
P35556	DA9	1142	F
P35556	DA9	1142	R
P35556	DA9	1156	F
P35556	DA9	1198	Y
P35556	DA9	1240	R
P35556	DA9	1246	F
P35556	DA9	1253	W
P35556	DA9	1253	Y
P35556	DA9	1257	W
P35556	DA9	1268	R
P35556	DA9	1384	F
P35556	DA9	1384	Y
P35556	DA9	1425	R
P35556	DA9	1434	S
P20933	AGU	163	S
P20933	AGU	306	R
P25445	ALPS1A	82	R
P07359	BSS	225	S
P04001	BCM	203	R
P04000	BCM	203	R
P21589	CALJA	358	Y
O75096	CLSS	160	Y
Q9UM47	CADASIL	43	G

Protein	Disease mnemonic	Disulfide bond position	Variant substitution
Q9UM47	CADASIL	49	F
Q9UM47	CADASIL	49	Y
Q9UM47	CADASIL	65	S
Q9UM47	CADASIL	67	Y
Q9UM47	CADASIL	76	R
Q9UM47	CADASIL	76	W
Q9UM47	CADASIL	87	R
Q9UM47	CADASIL	87	Y
Q9UM47	CADASIL	93	F
Q9UM47	CADASIL	93	Y
Q9UM47	CADASIL	106	W
Q9UM47	CADASIL	108	W
Q9UM47	CADASIL	108	Y
Q9UM47	CADASIL	117	F
Q9UM47	CADASIL	123	F
Q9UM47	CADASIL	123	Y
Q9UM47	CADASIL	128	Y
Q9UM47	CADASIL	134	W
Q9UM47	CADASIL	144	F
Q9UM47	CADASIL	144	S
Q9UM47	CADASIL	144	Y
Q9UM47	CADASIL	146	R
Q9UM47	CADASIL	155	S
Q9UM47	CADASIL	162	S
Q9UM47	CADASIL	174	F
Q9UM47	CADASIL	174	R
Q9UM47	CADASIL	174	Y
Q9UM47	CADASIL	183	F
Q9UM47	CADASIL	183	R
Q9UM47	CADASIL	183	S
Q9UM47	CADASIL	185	G
Q9UM47	CADASIL	185	R
Q9UM47	CADASIL	194	F
Q9UM47	CADASIL	194	R
Q9UM47	CADASIL	194	S
Q9UM47	CADASIL	194	Y
Q9UM47	CADASIL	201	Y

**Protein**	**Disease mnemonic**	**Disulfide bond position**	**Variant substitution**
Q9UM47	CADASIL	206	Y
Q9UM47	CADASIL	212	S
Q9UM47	CADASIL	222	G
Q9UM47	CADASIL	222	Y
Q9UM47	CADASIL	224	Y
Q9UM47	CADASIL	233	S
Q9UM47	CADASIL	233	Y
Q9UM47	CADASIL	240	S
Q9UM47	CADASIL	245	R
Q9UM47	CADASIL	251	R
Q9UM47	CADASIL	260	Y
Q9UM47	CADASIL	379	S
Q9UM47	CADASIL	395	R
Q9UM47	CADASIL	428	S
Q9UM47	CADASIL	428	Y
Q9UM47	CADASIL	440	G
Q9UM47	CADASIL	440	R
Q9UM47	CADASIL	446	S
Q9UM47	CADASIL	455	R
Q9UM47	CADASIL	484	F
Q9UM47	CADASIL	484	Y
Q9UM47	CADASIL	495	Y
Q9UM47	CADASIL	511	R
Q9UM47	CADASIL	542	Y
Q9UM47	CADASIL	549	Y
Q9UM47	CADASIL	775	S
Q9UM47	CADASIL	1015	R
Q9UM47	CADASIL	1261	R
Q9UM47	CADASIL	1261	Y
P50897	CLN1	45	Y
P50897	CLN1	152	Y
O14773	CLN2	365	R
O14773	CLN2	365	Y
P25189	CMT1B	50	
P04001	CBD	203	R
Q8IXK2	CRCS1	479	F
P06681	C2D	131	Y

Protein	Disease mnemonic	Disulfide bond position	Variant substitution
P02748	C9D	119	G
P00746	CFDD	214	R
P08603	CFHD	431	S
P08603	CFHD	536	R
P08603	CFHD	673	S
P08603	CFHD	959	Y
P27539	CTHM	267	Y
P98172	CFNS	153	S
P98172	CFNS	153	Y
P21802	CS	278	F
P21802	CS	278	Y
P21802	CS	342	F
P21802	CS	342	R
P21802	CS	342	S
P21802	CS	342	W
P21802	CS	342	Y
O95967	ARCL1B	267	Y
P25189	DSS	127	Y
P01185	NDI	52	R
P01185	NDI	58	F
P01185	NDI	59	R
P01185	NDI	59	Y
P01185	NDI	92	S
P01185	NDI	92	Y
P01185	NDI	98	G
P01185	NDI	98	S
P01185	NDI	104	F
P01185	NDI	104	G
P01185	NDI	105	R
P01185	NDI	105	Y
P01185	NDI	116	G
P01185	NDI	116	R
P01185	NDI	116	W
P16422	DIAR5	66	Y
Q9UNE0	ECTD10B	47	Y
Q9UNE0	ECTD10B	87	R
Q9UNE0	ECTD10B	148	R

**Protein**	**Disease mnemonic**	**Disulfide bond position**	**Variant substitution**
P20908	EDS1	1639	S
P16144	EB-PA	38	R
P16144	EB-PA	61	Y
P16144	EB-PA	245	G
P16144	EB-PA	562	R
P06280	FD	52	R
P06280	FD	52	S
P06280	FD	56	F
P06280	FD	56	G
P06280	FD	56	Y
P06280	FD	94	S
P06280	FD	94	Y
P06280	FD	142	R
P06280	FD	142	Y
P06280	FD	172	R
P06280	FD	172	Y
P06280	FD	202	W
P06280	FD	202	Y
P06280	FD	223	G
P06280	FD	378	Y
P08709	FA7D	82	F
P08709	FA7D	82	R
P08709	FA7D	121	F
P08709	FA7D	151	S
P08709	FA7D	195	R
P08709	FA7D	238	Y
P08709	FA7D	254	R
P08709	FA7D	254	Y
P08709	FA7D	370	F
P08709	FA7D	389	G
P00742	FA10D	149	Y
P00742	FA10D	151	Y
P00742	FA10D	390	F
P00742	FA10D	404	R
P03951	FA11D	46	F
P03951	FA11D	56	R
P03951	FA11D	140	Y

**Protein**	**Disease mnemonic**	**Disulfide bond position**	**Variant substitution**
P03951	FA11D	255	Y
P03951	FA11D	514	F
P00748	FA12D	590	S
P05160	FA13BD	450	F
P19438	FHF	59	R
P19438	FHF	59	S
P19438	FHF	62	G
P19438	FHF	62	Y
P19438	FHF	81	F
P19438	FHF	99	S
P19438	FHF	117	R
P19438	FHF	117	Y
P01130	FH	46	S
P01130	FH	89	Y
P01130	FH	95	G
P01130	FH	116	R
P01130	FH	134	F
P01130	FH	134	W
P01130	FH	160	Y
P01130	FH	173	W
P01130	FH	184	Y
P01130	FH	197	R
P01130	FH	222	Y
P01130	FH	231	G
P01130	FH	248	Y
P01130	FH	261	F
P01130	FH	276	R
P01130	FH	276	Y
P01130	FH	302	W
P01130	FH	302	Y
P01130	FH	313	Y
P01130	FH	318	F
P01130	FH	318	R
P01130	FH	329	F
P01130	FH	329	Y
P01130	FH	338	S
P01130	FH	358	Y

**Protein**	**Disease mnemonic**	**Disulfide bond position**	**Variant substitution**
P01130	FH	368	R
P01130	FH	379	Y
P01130	FH	667	Y
P01130	FH	677	R
P07911	HNFJ1	77	Y
P07911	HNFJ1	126	R
P07911	HNFJ1	148	W
P07911	HNFJ1	148	Y
P78363	FFM	1488	R
P04062	GD	55	S
P07602	AGD	388	F
Q07699	GEFS+1	121	W
P08514	GT	161	W
P08514	GT	705	R
P05106	GT	64	Y
P05106	GT	400	Y
P05106	GT	532	Y
P05106	GT	568	R
P05106	GT	586	F
P05106	GT	586	R
P05106	GT	601	R
Q99972	GLC1A	245	Y
Q99972	GLC1A	433	R
P10253	GSD2	103	G
P10253	GSD2	103	R
P10253	GSD2	108	G
P10253	GSD2	127	F
P07686	GM2G2	309	Y
P07686	GM2G2	534	Y
P17900	GM2GAB	138	R
Q30201	HFE1	282	Y
P81172	HFE2B	70	R
P81172	HFE2B	78	Y
P13987	HACD59	89	Y
P08603	AHUS1	325	Y
P08603	AHUS1	630	W
P08603	AHUS1	673	Y

**Protein**	**Disease mnemonic**	**Disulfide bond position**	**Variant substitution**
P08603	AHUS1	915	S
P08603	AHUS1	1043	R
P08603	AHUS1	1163	W
P15529	AHUS2	35	Y
P01024	AHUS5	1158	W
P00451	HEMA	172	W
P00451	HEMA	198	G
P00451	HEMA	267	Y
P00451	HEMA	348	R
P00451	HEMA	348	S
P00451	HEMA	348	Y
P00451	HEMA	1877	R
P00451	HEMA	1877	Y
P00451	HEMA	2040	Y
P00451	HEMA	2193	G
P00451	HEMA	2345	S
P00451	HEMA	2345	Y
P00740	HEMB	97	S
P00740	HEMB	102	R
P00740	HEMB	108	S
P00740	HEMB	119	F
P00740	HEMB	119	R
P00740	HEMB	134	Y
P00740	HEMB	155	F
P00740	HEMB	170	F
P00740	HEMB	178	R
P00740	HEMB	178	W
P00740	HEMB	252	S
P00740	HEMB	252	Y
P00740	HEMB	268	W
P00740	HEMB	382	Y
P00740	HEMB	396	F
P00740	HEMB	396	S
P00740	HEMB	407	R
P00740	HEMB	407	S
P00740	HEMB	435	Y
Q9BX67	HDBSCC	219	Y

Protein	Disease mnemonic	Disulfide bond position	Variant substitution
Q6UXH8	HLLS	174	R
P05155	HAE	130	Y
O95477	HDLD1	1477	R
P07949	HSCR1	142	S
P58335	HFS	218	R
P32004	HSAS	264	Y
P32004	HSAS	497	Y
P11362	HH2	101	F
P11362	HH2	178	S
P11362	HH2	277	Y
Q8N6C5	CHTE	942	R
P16473	CHNG1	41	S
P08842	IXL	446	Y
O14836	CVID2	104	R
P25942	HIGM3	83	R
O14836	IGAD2	104	R
P06213	IRAN type A	280	Y
Q8IU80	IRIDA	510	S
P01225	IFSHD	69	G
P21802	JWS	278	F
P21802	JWS	342	R
P21802	JWS	342	S
P36894	JPS	82	Y
P36894	JPS	124	R
P36894	JPS	130	R
P10912	LARS	56	S
P82279	LCA8	383	Y
P82279	LCA8	438	Y
P82279	LCA8	480	G
P82279	LCA8	480	R
P82279	LCA8	681	Y
P82279	LCA8	939	Y
P82279	LCA8	948	Y
P82279	LCA8	1174	G
P82279	LCA8	1218	F
P82279	LCA8	1321	S
P82279	LCA8	1332	F

**Protein**	**Disease mnemonic**	**Disulfide bond position**	**Variant substitution**
P06213	LEPRCH	301	Y
P60201	HLD1	220	Y
P60201	HLD1	228	Y
P15289	MLD	156	R
P15289	MLD	172	Y
P15289	MLD	300	F
P15289	MLD	489	G
P07602	MLD-SAPB	241	S
O00584	LCWM	184	R
Q13326	LGMD2C	283	Y
P06858	LPL deficiency	243	S
P06858	LPL deficiency	266	W
P06858	LPL deficiency	305	R
P06858	LPL deficiency	310	Y
P06858	LPL deficiency	445	Y
P26842	LPFS2	53	Y
P55000	MDM	77	R
P55000	MDM	99	Y
O00754	MANSA	55	F
O00754	MANSA	501	S
P35555	MFS	89	F
P35555	MFS	111	R
P35555	MFS	123	Y
P35555	MFS	129	Y
P35555	MFS	154	S
P35555	MFS	166	F
P35555	MFS	166	S
P35555	MFS	177	R
P35555	MFS	476	G
P35555	MFS	499	Y
P35555	MFS	504	F
P35555	MFS	541	Y
P35555	MFS	570	Y
P35555	MFS	587	Y
P35555	MFS	596	Y
P35555	MFS	598	W
P35555	MFS	611	R

**Protein**	**Disease mnemonic**	**Disulfide bond position**	**Variant substitution**
P35555	MFS	617	G
P35555	MFS	628	K
P35555	MFS	652	S
P35555	MFS	734	F
P35555	MFS	748	Y
P35555	MFS	750	G
P35555	MFS	776	G
P35555	MFS	776	Y
P35555	MFS	781	R
P35555	MFS	781	Y
P35555	MFS	790	Y
P35555	MFS	811	Y
P35555	MFS	816	S
P35555	MFS	832	Y
P35555	MFS	853	S
P35555	MFS	862	R
P35555	MFS	890	G
P35555	MFS	890	R
P35555	MFS	908	R
P35555	MFS	921	G
P35555	MFS	926	R
P35555	MFS	926	Y
P35555	MFS	1044	Y
P35555	MFS	1053	R
P35555	MFS	1055	G
P35555	MFS	1055	W
P35555	MFS	1055	Y
P35555	MFS	1068	G
P35555	MFS	1074	R
P35555	MFS	1086	W
P35555	MFS	1117	G
P35555	MFS	1117	Y
P35555	MFS	1129	Y
P35555	MFS	1153	S
P35555	MFS	1153	Y
P35555	MFS	1171	W
P35555	MFS	1223	Y

Protein	Disease mnemonic	Disulfide bond position	Variant substitution
P35555	MFS	1242	Y
P35555	MFS	1249	S
P35555	MFS	1265	R
P35555	MFS	1278	S
P35555	MFS	1284	G
P35555	MFS	1284	Y
P35555	MFS	1333	S
P35555	MFS	1339	Y
P35555	MFS	1350	F
P35555	MFS	1374	S
P35555	MFS	1389	R
P35555	MFS	1402	R
P35555	MFS	1429	S
P35555	MFS	1431	W
P35555	MFS	1431	Y
P35555	MFS	1513	R
P35555	MFS	1564	F
P35555	MFS	1564	Y
P35555	MFS	1589	F
P35555	MFS	1610	G
P35555	MFS	1631	G
P35555	MFS	1663	R
P35555	MFS	1663	Y
P35555	MFS	1770	F
P35555	MFS	1791	R
P35555	MFS	1791	Y
P35555	MFS	1793	W
P35555	MFS	1806	S
P35555	MFS	1806	Y
P35555	MFS	1833	S
P35555	MFS	1835	Y
P35555	MFS	1876	Y
P35555	MFS	1895	R
P35555	MFS	1900	Y
P35555	MFS	1928	G
P35555	MFS	1928	R
P35555	MFS	1928	Y

**Protein**	**Disease mnemonic**	**Disulfide bond position**	**Variant substitution**
P35555	MFS	1934	S
P35555	MFS	1971	Y
P35555	MFS	1977	Y
P35555	MFS	1984	R
P35555	MFS	1998	Y
P35555	MFS	2038	Y
P35555	MFS	2085	R
P35555	MFS	2099	W
P35555	MFS	2111	R
P35555	MFS	2111	Y
P35555	MFS	2142	Y
P35555	MFS	2151	W
P35555	MFS	2221	F
P35555	MFS	2221	G
P35555	MFS	2221	S
P35555	MFS	2251	R
P35555	MFS	2258	R
P35555	MFS	2307	S
P35555	MFS	2406	Y
P35555	MFS	2442	S
P35555	MFS	2442	W
P35555	MFS	2489	R
P35555	MFS	2500	R
P35555	MFS	2500	Y
P35555	MFS	2511	R
P35555	MFS	2535	W
P35555	MFS	2571	R
P35555	MFS	2581	F
P35555	MFS	2592	S
P35555	MFS	2605	R
P35555	MFS	2605	Y
P35555	MFS	2646	R
P35555	MFS	2652	G
P35555	MFS	2663	S
O00187	MASPD	156	CHNH
P07911	MCKD2	148	W
P15260	MSMD	77	Y

Protein	Disease mnemonic	Disulfide bond position	Variant substitution
P24043	MDC1A	862	R
O60220	MTS	66	W
Q3T906	MLII	461	G
Q3T906	MLIIIA	461	G
P15848	MPS6	117	R
P15848	MPS6	192	R
P15848	MPS6	405	Y
P15848	MPS6	521	Y
P49747	EDM1	348	F
P49747	EDM1	371	S
P49747	EDM1	371	Y
P49747	EDM1	410	Y
P36897	MSSE	41	Y
Q8NBK3	MSD	218	Y
Q96KG7	EMARDD	326	R
Q96KG7	EMARDD	774	R
Q2I0M5	NDNC4	95	F
Q2I0M5	NDNC4	107	R
Q2I0M5	NDNC4	118	Y
O60500	NPHS1	265	R
O60500	NPHS1	417	F
O60500	NPHS1	465	Y
O60500	NPHS1	528	F
O60500	NPHS1	623	F
P17405	NPDB	92	W
P17405	NPDB	157	R
P17405	NPDB	431	R
O15118	NPC1	63	R
O15118	NPC1	74	Y
O15118	NPC1	113	R
O15118	NPC1	177	G
O15118	NPC1	177	Y
O15118	NPC1	247	Y
P61916	NPDC2	47	F
P61916	NPDC2	93	F
P61916	NPDC2	99	R
O15303	CSNB1B	522	Y

Protein	Disease mnemonic	Disulfide bond position	Variant substitution
Q3SXY7	CSNB1F	328	Y
Q00604	ND	39	R
Q00604	ND	55	R
Q00604	ND	65	W
Q00604	ND	65	Y
Q00604	ND	69	S
Q00604	ND	96	W
Q00604	ND	96	Y
Q00604	ND	110	G
Q00604	ND	110	R
Q00604	ND	126	S
Q00604	ND	128	R
P08123	OI1	1195	Y
Q9Y6Q6	OPTB7	175	R
P07477	PCTT	139	F
P53634	PLS	291	Y
P21802	PS	278	F
P21802	PS	342	G
P21802	PS	342	R
P21802	PS	342	S
P21802	PS	342	Y
P55268	PIERSS	321	R
P02458	PLSD-T	1485	G
P98161	PKD1	436	R
P98161	PKD1	508	R
P03952	PKK deficiency	548	Y
P49747	PSACH	328	R
P49747	PSACH	348	R
P49747	PSACH	387	G
P49747	PSACH	387	R
P49747	PSACH	410	Y
P49747	PSACH	448	S
P49747	PSACH	468	Y
Q13873	PPH1	117	Y
P82279	RP12	45	W
P82279	RP12	157	S
P82279	RP12	195	F

**Protein**	**Disease mnemonic**	**Disulfide bond position**	**Variant substitution**
P82279	RP12	250	W
P82279	RP12	891	G
P82279	RP12	948	Y
P82279	RP12	1165	W
P82279	RP12	1174	G
P82279	RP12	1181	R
Q5T1H1	RP25	1176	R
Q5T1H1	RP25	2139	Y
Q5T1H1	RP25	2911	Y
O75445	RP39	759	F
P08100	RP4	110	F
P08100	RP4	110	Y
O15537	XLRS1	110	Y
O15537	XLRS1	142	W
O15537	XLRS1	219	G
O15537	XLRS1	219	R
Q01974	RRS	182	Y
Q9BQB4	SOST1	167	R
P31785	XSCID	62	G
P31785	XSCID	115	F
P31785	XSCID	115	R
P31785	XSCID	182	R
P31785	XSCID	231	Y
P35555	SGS	1223	Y
O15232	SEMD-MATN3	304	S
P78363	STGD1	75	G
P78363	STGD1	1488	F
P78363	STGD1	1488	R
P78363	STGD1	1488	Y
P35555	SSKS	1564	S
P35555	SSKS	1577	G
Q13253	SYM1A	184	Y
P37023	HHT2	51	Y
P37023	HHT2	77	W
P04196	THPH11	241	R
P04070	THPH3	426	Y
P07225	THPH5	121	Y

Protein	Disease mnemonic	Disulfide bond position	Variant substitution
P07225	THPH5	161	G
P07225	THPH5	175	F
P07225	THPH5	186	Y
P07225	THPH5	241	S
P07225	THPH5	247	G
P07225	THPH5	265	R
P07225	THPH5	265	W
P07225	THPH5	267	S
P07225	THPH5	449	S
P07225	THPH5	475	R
P07225	THPH5	639	F
P07225	THPH5	639	Y
P07225	THPH5	666	R
Q76LX8	TTP	311	Y
Q76LX8	TTP	347	S
P07202	TDH2A	808	R
P01266	TDH3	183	Y
O75445	USH2A	536	R
O75445	USH2A	575	S
O75445	USH2A	759	F
Q96GP6	VDEGS	258	Y
O75197	EVR4	1253	F
O75197	EVR4	1361	G
P04275	VWD1	1149	R
P04275	VWD2	1060	R
P04275	VWD2	1272	F
P04275	VWD2	1272	R
P04275	VWD3	2739	Y
